# Different applications of the KDIGO criteria for AKI lead to different incidences in critically ill patients: a post hoc analysis from the prospective observational SICS-II study

**DOI:** 10.1186/s13054-020-02886-7

**Published:** 2020-04-21

**Authors:** Renske Wiersema, Sakari Jukarainen, Ruben J. Eck, Thomas Kaufmann, Jacqueline Koeze, Frederik Keus, Ville Pettilä, Iwan C. C. van der Horst, Suvi T. Vaara

**Affiliations:** 1 Department of Critical Care, University Medical Center Groningen, University of Groningen, Groningen, The Netherlands; 2 Division of Intensive Care Medicine, Department of Anesthesiology, Intensive Care and Pain Medicine, University of Helsinki and Helsinki University Hospital, Helsinki, Finland; 3 Department of Internal Medicine, University Medical Center Groningen, University of Groningen, Groningen, The Netherlands; 4 Department of Anaesthesiology, University Medical Center Groningen, University of Groningen, Groningen, The Netherlands; 5 Department of Intensive Care, Maastricht University Medical Center+, Maastricht University, Maastricht, The Netherlands

**Keywords:** Acute kidney injury, Heterogeneity, Critically ill, Epidemiology, Mortality, Randomized controlled trials

## Abstract

**Background:**

Acute kidney injury (AKI) is a frequent and clinically relevant problem in critically ill patients. Various randomized controlled trials (RCT) have attempted to assess potentially beneficial treatments for AKI. Different approaches to applying the Kidney Disease Improving Global Outcomes (KDIGO) criteria for AKI make a comparison of studies difficult. The objective of this study was to assess how different approaches may impact estimates of AKI incidence and whether the association between AKI and 90-day mortality varied by the approach used.

**Methods:**

Consecutive acutely admitted adult intensive care patients were included in a prospective observational study. AKI was determined following the KDIGO criteria during the first 7 days of ICU admission. In this post hoc analysis, we assessed whether AKI incidence differed when applying the KDIGO criteria in 30 different possible methods, varying in (A) serum creatinine (sCr), (B) urine output (UO), and (C) the method of combining these two into an outcome, e.g., severe AKI. We assessed point estimates and 95% confidence intervals for each incidence. Univariable regression was used to assess the associations between AKI and 90-day mortality.

**Results:**

A total of 1010 patients were included. Baseline creatinine was available in 449 (44%) patients. The incidence of any AKI ranged from 28% (95%CI 25–31%) to 75% (95%CI 72–77%) depending on the approach used. Methods to estimate missing baseline sCr caused a variation in AKI incidence up to 15%. Different methods of handling UO caused a variation of up to 35%. At 90 days, 263 patients (26%) had died, and all 30 variations were associated with 90-day mortality.

**Conclusions:**

In this cohort of critically ill patients, AKI incidence varied from 28 to 75%, depending on the method used of applying the KDIGO criteria. A tighter adherence to KDIGO definitions is warranted to decrease the heterogeneity of AKI and increase the comparability of future studies.

## Background

Acute kidney injury (AKI) is a highly complex and common syndrome associated with increased mortality. A myriad of ways to report the incidence of AKI has been proposed [1]. The first diagnostic criteria for AKI were created to reduce heterogeneity in reporting AKI and outcomes; the Risk, Injury, Failure, Loss of kidney function, and End-stage kidney disease (RIFLE) classification [2], which were slightly updated into the Acute Kidney Injury Network (AKIN) classification [3]. The Kidney Disease Improving Global Outcomes (KDIGO) criteria combine these and are currently recommended to assess AKI [4]. The KDIGO definition relies on three diagnostic criteria: a rise in serum creatinine (sCr), a decrease in urine output (UO), and administration of renal replacement therapy (RRT) [4].

Despite efforts to unify the diagnosis and reporting of AKI [5], recent literature has reported varying AKI incidences and outcomes [6, 7]. Multiple ways of applying the KDIGO criteria exist. Some limitations are inherent to the physiological basis of the criteria themselves. For example, using sCr has its limitations, such as a delay in the rise after an insult, and fluctuations according to fluid and nutritional status as well as muscle mass [8]. Moreover, a baseline sCr value is required for the comparison of sCr measurements during admission, which is often lacking in acutely admitted critically ill patients [9, 10]. Various formulas to estimate baseline sCr exist, which lead to varying AKI incidences and misclassification of AKI [9–13]. The criteria for UO are less frequently used as it is challenging to collect hourly UO data prospectively, and electronic health record data may be unreliable [14]. Using only the sCr criteria, however, likely underestimates AKI incidence [15–17].

Besides the limitations inherent to sCr and UO in critically ill patients, the KDIGO criteria are currently applied in different ways leading to various incidences and outcomes, due to different interpretations but also using modifications to KDIGO to fit the data available. For example, the various formulas for baseline sCr and UO, calculating the average UO per kilogram per hour using a 6-h time interval is theoretically more sensitive for detecting AKI compared to 24-h intervals [4].

AKI is a significant clinical problem and at the core of many ongoing research efforts. Randomized controlled trials (RCTs) investigating different aspects of AKI in the last 5 years have used various criteria as either inclusion or outcome definitions, and this may hamper the comparison of these RCTs (Additional file 1: E-Table 1. Examples of RCTs). A more standardized and therewith uniform approach towards applying KDIGO criteria may aid in decreasing the variety in used definitions for AKI, to help further increase the comparability of future trials. In this study, we aimed to investigate whether and how different applications of methods affect AKI outcomes depending on the options of handling (A) (baseline) sCr, (B) UO data, and (C) reported outcomes.

## Methods

The Simple Intensive Care Studies II (SICS-II) was a single-center, prospective observational study designed to evaluate the diagnostic and prognostic value of repeated clinical examination and ultrasonography in critically ill patients (NCT03577405) [18].

### Objective

The primary objective of this post hoc analysis was to evaluate how the options for applying the KDIGO criteria influence the incidence of AKI.

**Fig. 1:**
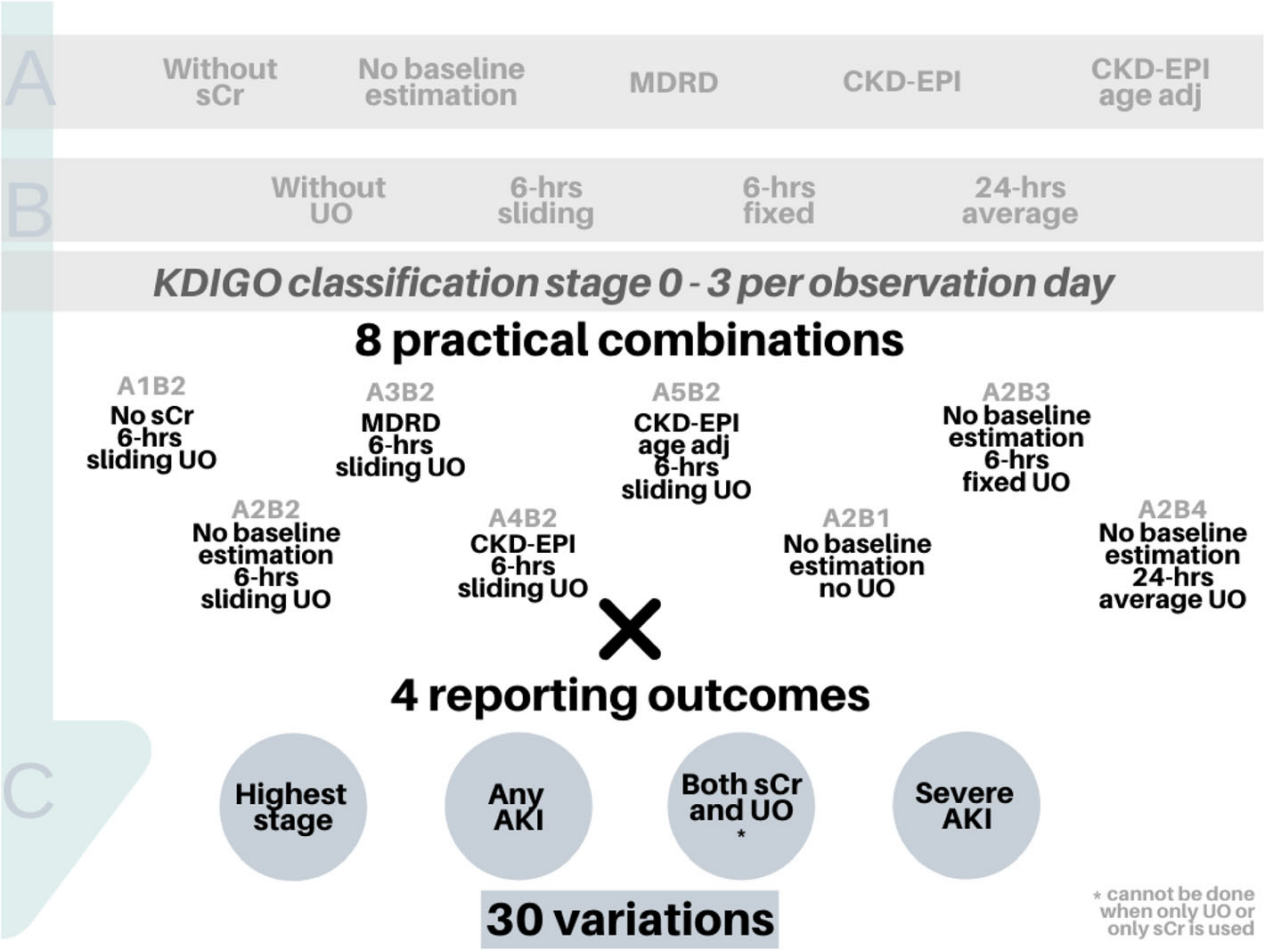
Different options for defining and reporting AKI outcomes. Illustration of how different theoretical options in serum creatinine (level A, five variants) and urine output (level B, four variants) could lead to twenty different ways of assigning a KDIGO stage per observation day. Here, eight practical combinations of A and B are shown. In total, this results in a total of 32 variations of reporting AKI, as AKI can be expressed for example using one of the four displayed reporting outcomes. However, defining AKI on both sCr and UO cannot be done for the two practical combinations in which either sCr or UO is used. Hence, 30 different variations were investigated. AKI, acute kidney injury; sCr, serum creatinine; UO, urine output; MDRD, Modification of Diet in Renal Disease; CKD-EPI, Chronic Kidney Disease Epidemiology Collaboration

We evaluated different options in terms of the use of (A) serum creatinine, (B) urine output, and (C) the method of reporting the AKI outcomes (Fig. 1). Renal replacement therapy (RRT) was handled similarly in every variant, always resulting in KDIGO stage 3 for that observation day.

#### Options for serum creatinine (A)

In theory, AKI can be assessed without using sCr (A1). If serum creatinine is used, there are various methods to estimate the sCr baseline if this is unknown. Four variations are suggested for estimating the baseline sCr. First, without any formula, the first sCr observation is used as a reference value if this is missing (A2) [19, 20]. Second, while assuming a clearance of 75 mL/min/m^2^, the Modification of Diet in Renal Disease (MDRD) formula (A3) [21], the Chronic Kidney Disease Epidemiology Collaboration (CKD-EPI) formula without age adjustment (A4) [22], or the CKD-EPI formula with age-adjusted GFR (A5) [12] can be used to estimate the baseline sCr if this is missing.

#### Options for the use of urine output (B)

UO is frequently not considered in (notably retrospective) AKI studies (B1). When UO is taken into consideration, there are at least three methods to apply UO criteria: using hourly registered data for 6-h windows (sliding method; B2) or fixed 6-h windows (fixed method; B3) (Additional file 1: E-figure 1), or using 24-h cumulative UO samples divided by four (B4) to assess the average UO for 6 h.

#### Options for reporting AKI outcomes (C)

Four different methods have been suggested for reporting the incidence of AKI. The first method is without reclassification, so essentially reporting KDIGO AKI stages, thus reporting the highest observed stage during the observation period (C1) [6]. The second is a reclassification of a categorical KDIGO variable into a dichotomous variable where no AKI includes AKI stage 0, and any AKI includes AKI stage 1, stage 2, or stage 3 (C2) [23]. The third method is equal to the second method but requests that both sCr and UO criteria (in contrast with either sCr or UO criteria) are fulfilled (C3) [15]. The fourth method is only severe AKI, where no severe AKI is any AKI stage 0 or 1, and severe AKI is any AKI stage 2 or stage 3 (C4) [24]. More granular methods that also include an aspect of time, such as persistent AKI [25, 26], the duration of AKI [27], or AKI burden [28] (a proportion of severity and duration based on available data), were not included in the primary analysis but presented as supplements.

### Theory versus practice

Theoretically, in total, 80 possible combinations can be calculated from how to apply the different options (Fig. 1). In practice, however, some options are clinically somewhat irrelevant. For example, creatinine is nearly always used (i.e., option A1 might be clinically irrelevant), some studies advise only to use available baseline sCr (A2) and using 6-h sliding windows for UO (B2). In this study, we therefore chose to calculate the frequently applied methods, i.e., to use 6-h sliding windows when testing sCr options, and to assume no baseline estimation when combining with options of UO. Together, variations in sCr and UO then assemble eight options. Multiplying these eight options of variations in A and B by four options of reporting AKI results in 32 sensible variations. However, for C3, it is not possible to calculate this without either sCr (A1) or UO (B1). Thus, in total, we calculated 30 sensible variations of AKI (i.e., A2B2C4, indicating AKI based on sCr without baseline estimation, and UO based on sliding 6-h windows, expressed as severe AKI).

### Participants

All patients admitted to the ICU were screened 24/7 for eligibility during the study period. The target population included acutely admitted patients above 18 years of age, with an expected ICU stay of at least 24 h. Patients were excluded if they were previously included in this study, if they were in strict isolation limiting patient access for research purposes, or when informed consent was not obtained. The local institutional review board approved the study (M18.228393). In patients not capable of providing consent due to the acute illness, informed consent was first obtained from the legal representatives. Consent for the use of the study data was asked at a later time whenever possible. If the patient deceased before consent was obtained, the study data was used, and the legal representatives were informed of the study.

### Variables

We registered patient characteristics such as demographical data, comorbidities, and severity of illness scores at admission. Comorbidity data were defined following the Dutch National Intensive Care Evaluation (NICE) registry; specifically, chronic kidney disease (CKD) was defined by serum creatinine above 177 μmol/L [29, 30]. Patient evaluations included clinical examination and ultrasound, which were performed within 3 and 24 h of ICU admission, respectively. Measurements were conducted by research interns and PhD students, who were not involved in patient care. Data for AKI diagnosis were extracted from electronic health records (EHR). All available data on sCr, UO, and RRT were collected during the first 7 days of ICU admission. Baseline sCr was defined as the lowest value of sCr in the year before ICU admission. We assessed whether any baseline sCr value was available (up to 1 year prior to ICU admission) in the EHR which could be from appointments with specialists or previous admissions. UO data were extracted from the EHR in two ways which were separately analyzed: the fluid registry as filled in by health care providers at the bedside which was used for options B2 and B3; 24-h urine collection samples sent for laboratory analysis as part of routine daily care (option B4). Outcomes were assessed as AKI incidence and 90-day mortality. The 90-day mortality data were obtained from the municipal registry. If patients emigrated within the 90-day follow-up period, they were considered lost to follow-up.

### Statistics

Continuous variables were reported as means (with standard deviations (SD)) or medians (with interquartile ranges (IQR)) depending on the distributions. Categorical data, including AKI incidences, were presented in proportions. We evaluated the different incidences that resulted from the various combinations and calculated the 95%CIs.

Associations with 90-day mortality were assessed with univariable logistic regression analysis. The area under the ROC curve was assessed as a measure of the performance of each variant to predict mortality. *P* values of < 0.05 were considered statistically significant. Analyses were performed using Stata version 15 (StataCorp, College Station, TX, USA) and R version 3.5.1.

### Sensitivity analyses

We performed two sensitivity analyses. First, we repeated our analysis after excluding patients with CKD to assess whether this would change our results. Second, we repeated our analysis after excluding patients with unknown baseline sCr.

## Results

Between May 14, 2018, and July 10, 2019, a total of 3357 patients were assessed for eligibility, of whom 1104 fulfilled the inclusion criteria. Data were not obtained for 94 patients; as 45 patients died before inclusion, continuous resuscitation efforts were made in 26 patients and for other logistic reasons in 23 patients. In total, 1010 patients (91% of 1104) were included in the SICS-II cohort (Table 1, Additional file 1: E-Figure 2).

**Table 1:** Baseline characteristics of included patients

	*N* = 1010
Age, years (SD)	61 (15)
Gender, male, *n* (%)	630 (62%)
Admission type, %	
Medical	64%
Acute surgery	32%
Other*	4%
BMI, kg/m^2^ (SD)	26 (5)
Diabetes mellitus, *n* (%)	190 (19%)
Liver cirrhosis, *n* (%)	43 (4%)
APACHE IV, mean (SD)	70 (31)
Chronic kidney disease, *n* (%)	88 (9%)
Observed baseline serum creatinine, mmol/L (IQR)	76 (58, 102)
At study inclusion	
Mechanical ventilation, *n* (%)	530 (52%)
Use of vasopressors, *n* (%)	458 (45%)
Use of RRT, *n* (%)	61 (6%)
Glasgow coma scale, (IQR)	9 (3, 15)
Respiratory rate, breaths per minute (SD)	18 (6)
Systolic blood pressure, mmHg (SD)	118 (28)
Central temperature, °C (SD)	36.8 (1.2)
Urine output at inclusion, mL/kg/h (IQR)	0.7 (0.2, 1.7)
Outcomes	
ICU length of stay, days (IQR)	2 (1, 5)
ICU mortality, *n* (%)	165 (16%)
90-day mortality, *n* (%)	263 (26%)

*SD* standard deviation, *RRT* renal replacement therapy, *APACHE* Acute Physiology and Chronic Health Evaluation, *IQR* interquartile range, *ICU* intensive care unit

*Other, for example, unplanned admissions after planned surgery due to an adverse event

### Data availability

In 449 patients (44%), the sCr baseline was registered, and for the other 561 patients, the sCr baseline was estimated using the various options (Additional file 1: E-Table 4). Among patients with estimated baseline sCr, the median first sCr was 90 (IQR 69–122) μmol/L. The median observation period was 2 days (IQR 1–5). Of the 1010 patients, 658 patients (65%) were discharged to the ward, and 135 patients (13%) died during the first 7 days of ICU admission. Eighty-eight patients (9%) had CKD. Altogether, 34 patients (3%) were treated with dialysis before admission, and in 25 of them, a baseline Cr was known. In 1008 patients (99%), one or more sCr measurements were available during the first 7 days of ICU admission. RRT was instigated in 61 patients (6%) during the first 7 days of ICU admission. One or more 24-h urine collection period samples were available for 728 patients (72%). UO data in hourly samples were available for 989 patients (98%); the median number of hours available was 47 h (IQR 22–120), with the median percentage of hours available out of ICU stay hours being 91% (76–96%). In 15 patients (1.5%), no data on UO were available. Missing data per calendar day are shown in Additional file 1: E-Table 5.

### Acute kidney injury

**Fig. 2:**
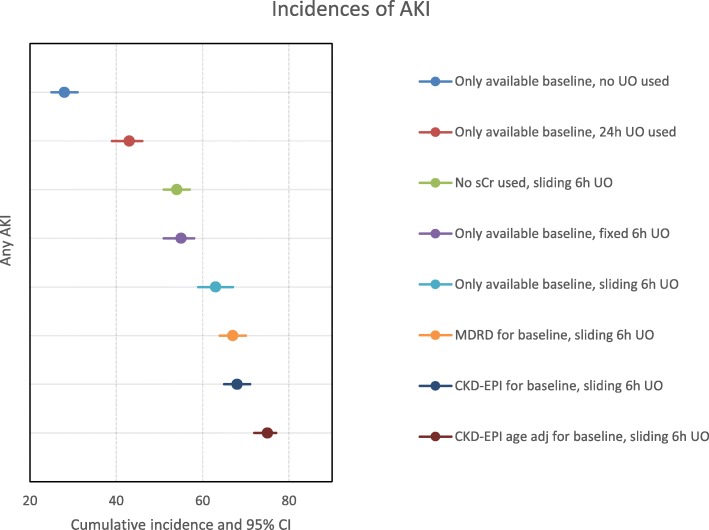
Variation in incidence for diagnosis of acute kidney injury (AKI) according to KDIGO using the same data from the same study population (*N* = 1010). Illustration of how different methods in terms of sCr and UO cause variation in the cumulative incidence of any AKI (reporting method C2). AKI, acute kidney injury; sCr, serum creatinine; UO, urine output, MDRD, Modification of Diet in Renal Disease; CKD-EPI, Chronic Kidney Disease Epidemiology Collaboration

Overall, AKI incidence ranged from 28% (95%CI 25–31%) to 75% (95%CI 72–77%) when applying the 30 different approaches to AKI (Fig. 2, Additional file 1: E-Table 2). Incidence was highest when using the age-adjusted CKD-EPI formula to estimate the baseline sCr and sliding 6-h UO windows. Incidences were lowest when using only available baseline sCr and no estimation formula, combined with 24-h cumulative UO values or without using UO at all. Different sCr options caused a variation of up to 15%. Different UO approaches caused a variation of up to 35%. More granular methods, like AKI burden similarly, varied (Additional file 1: E-Table 3).

### Ninety-day mortality

Eleven patients (1%) were lost to follow-up due to migration. At the 90-day follow-up, 263 patients (26%) had died (Table 1). Most applied criteria for AKI were associated with 90-day mortality (Additional file 1: E-Table 6). Of all different criteria, using no baseline creatinine estimation combined with 24-h cumulative UO data seemed to have the strongest univariable association with 90-day mortality for AKI burden and using no UO data at all for persistent AKI (Additional file 1: E-Table 7).

### Sensitivity analyses

Variation of incidences was similar when excluding patients with CKD (Additional file 1: E-Fig. 3). Variation in AKI incidence was 26% when including only patients in whom baseline sCr was known (Additional file 1: E-Fig. 4).

## Discussion

In this post hoc study using data from a prospective observational study on AKI in acutely admitted critically ill patients, we showed that AKI incidence varied depending on the method used to apply KDIGO criteria. Our results may partly explain the high variability in AKI incidence in the literature and provide an opportunity to increase the comparability for future observational studies and RCTs focusing on AKI.

Previous studies have assessed the differences between previous versions of AKI definitions such as the RIFLE criteria and demonstrated that these differences led to varying associations between AKI and outcomes [31, 32]. The initiative to define AKI uniformly was based on the need to be able to compare research results. Still, based on our hypothesis, up to 80 different methods can be used to assess AKI using the current KDIGO definition. Methods varied based on baseline sCr handling, UO data handling, how AKI is defined (any AKI, severe AKI, etc.), and whether both UO and sCr should be fulfilled or either [33], as it is recommended they are to be used independently for diagnosis and staging. In conclusion, without one uniform approach to KDIGO criteria, comparison of study outcomes or systematic reviews of previous AKI studies is complicated.

The impact on the incidence of AKI using different methods to enter baseline sCr has been widely recognized [20, 34]. The absence of a sCr baseline and differences in methods handling the missing data should be standardized, as back estimations seem insufficient [12], and in a sensitivity analysis, we showed that variation of AKI incidence decreased if baseline sCr was known and thus no estimation was needed. Overall, it seems from our results that using only available baseline sCr and no estimation results for sCr leads to the strongest association with mortality. One possible explanation for this finding could be that the patients with a measured sCr baseline have a reason to be hospitalized or even had renal failure. However, we deem this explanation unlikely, as the observed sCr values are within normal ranges.

Even though most recent studies have used the complete KDIGO criteria, many studies discard or change the UO criteria, despite that adding UO significantly increases AKI incidence [15–17]. Obtaining UO data may be challenging, as optimally hourly UO is recorded. Registration may be inaccurate, and missed observations can mistakenly be registered as anuria with implications for AKI diagnosis. Additionally, the KDIGO does not take ideal body weight into account, and often, only weight at admission or an estimation is used, influencing AKI diagnosis [35, 36]. Notably, the incidence of AKI was higher when using a sliding window for 6-h periods, indicating that each hour could mean that the average UO of the previous 6 h adjusts to below 0.5 mL/kg/h, logically increasing AKI incidence. Further investigation regarding the use of sliding 6-h UO as AKI criterion is needed, elucidating whether the 6-h UO should be measured consecutively or averaged to determine whether a patient has oliguria.

Some of the approaches illustrated in this manuscript are a result of a difference in interpretation of the KDIGO criteria, while others are intended modifications applied to fit certain studies or to assess its prognostic values. Nonetheless, all the various methods that are currently used to express AKI were likely adapted to better appreciate AKI heterogeneity. However, the resulting variation in research complicates the generalizability of results and may profoundly bias some conclusions.

### Strengths and limitations

Some strengths of this study exist. First, it was performed following a pre-published protocol, and almost all eligible patients were included. Second, instead of choosing one method to define AKI to answer the original research questions, we chose to perform a post hoc analysis to evaluate the variability in results. Some apparent limitations need to be considered. First, as our data were to some extent incomplete, we, thus, only performed a complete case analysis for the different definitions of AKI. Therefore, not all 30 approaches could be compared in the entire population. Second, some data were lacking throughout the seven observation days, as patients could have been discharged to the ward or deceased during this period, and if less than 24-h data were available, no KDIGO stage 3 could be diagnosed based on UO. However, the amount of missing data while patients were still in the ICU was low, and patients with an expected stay below 24 h were excluded. Third, we included an all-comers population of critically ill patients, and therefore, studying subgroups, such as patients with sepsis, could have further explicated our results. However, we consider including a heterogeneous group of patients as appropriate to illustrate the existence and magnitude of the problem. Fourth, this was a single-center study, and the lack of external validation is an important limitation. Last, our outcome was 90-day mortality, and we did not assess the development of organ dysfunction, administration of RRT, or any patient-reported outcomes. These outcomes could, combined with a longer follow-up, potentially aid in identifying clinically relevant phenotypes of AKI.

## Conclusion

In this cohort of critically ill patients, AKI incidence varied from 28 to 75%, depending on the method used to apply the same KDIGO definition. Availability of baseline sCr decreased incidence variation. A more uniform application of the KDIGO definitions for AKI could decrease the variety of AKI incidences and increase the comparability of future studies.

## Supplementary information

**Additional file 1: E-Table 1.** Example of RCT’s with either KDIGO AKI as outcome or KDIGO AKI as inclusion criteria. **E-Table 2.** Different incidences of acute kidney injury according to 30 different methodological options in the same population of critically ill patients. **E-Table 3.** Different incidences of acute kidney injury with more granular methods. **E-Table 4.** Estimated and actual baseline creatinine. **E-Table 5.** Missing data by calendar day. **E-Table 6.** Univariable associations with 90-day mortality, odds ratios per AKI variation. **E-Table 7.** Univariable associations with 90-day mortality from more granular methods. **E-Figure 1.** Example of using fixed versus sliding 6 hour windows vs actual (simulated) UO. **E-Figure 2.** Flowchart of study inclusion. **E-Figure 3.** Variation in AKI incidence after excluding CKD patients. **E-Figure 4.** Variation in AKI incidence after excluding patients where no baseline sCr was known.

## Data Availability

The dataset used and/or analyzed during the current study are available from the corresponding author on reasonable request.

## Abbreviations

AKI: Acute kidney injury
AKIN: Acute Kidney Injury Network
APACHE IV: Acute Physiology and Chronic Health Evaluation
AUROC: Area under the receiver operating curve
CI: Confidence interval
CKD-EPI: Chronic Kidney Disease Epidemiology Collaboration
ESM: Electronic supplemental material
KDIGO: Kidney Disease Improving Global Outcome
MDRD: Modification of Diet in Renal Disease
OR: Odds ratio
ICU: Intensive care unit
IQR: Interquartile range
RCT: Randomized controlled trial
RIFLE: Risk, Injury to the kidney, Failure of kidney function, Loss of kidney function, and End-stage kidney disease
RRT: Renal replacement therapy
sCr: Serum creatinine
SD: Standard deviation
SICS-II: Simple Intensive Care Studies II
UMCG: University Medical Centre Groningen
UO: Urine output
