# Supplementary material for: Different applications of the KDIGO criteria for AKI lead to different incidences in critically ill patients: a post hoc analysis from the prospective observational SICS-II study
Source: Crit Care. 2020 Apr 21;24:164. doi: 10.1186/s13054-020-02886-7 (PMC7175574; doi:10.1186/s13054-020-02886-7)
Supplement: Supplementary file 1 — Additional file 1: E-Table 1. Example of RCT’s with either KDIGO AKI as outcome or KDIGO AKI as inclusion criteria. E-Table 2. Different incidences of acute kidney injury according to 30 different methodological options in the same population of critically ill patients. E-Table 3. Different incidences of acute kidney injury with more granular methods. E-Table 4. Estimated and actual baseline creatinine. E-Table 5. Missing data by calendar day. E-Table 6. Univariable associations with 90-day mortality, odds ratios per AKI variation. E-Table 7. Univariable associations with 90-day mortality from more granular methods. E-Figure 1. Example of using fixed versus sliding 6 hour windows vs actual (simulated) UO. E-Figure 2. Flowchart of study inclusion. E-Figure 3. Variation in AKI incidence after excluding CKD patients. E-Figure 4. Variation in AKI incidence after excluding patients where no baseline sCr was known. [file 13054_2020_2886_MOESM1_ESM.docx]

**E-Table 1.** Example of RCT’s with either KDIGO AKI as outcome or KDIGO AKI as inclusion criteria

| First author, year  (Trial name) | Center, #pt | Population | Study objective | Serum creatinine  methods | Urine output  methods | Reported in  terms of |
| --- | --- | --- | --- | --- | --- | --- |
| AKI as outcome | | | | | | |
| Young, P.  2015 [1]  (SPLIT) | 4, 2778 | Adult ICU patients requiring crystalloid fluid therapy | Crystalloid Solution vs Saline on Acute Kidney Injury | Did not estimate baseline sCr if this was missing, further according to KDIGO | No urine output used | Any AKI |
| Barba-Navarro, R.  2017 [2] | 1, 233 | Adult patients after elective or emergent cardiac surgery requiring CPB | Spironolactone in CSA-AKI | Baseline sCr was value from 1 day prior to surgery, further according to KDIGO | Urine output was measured every shift | Any AKI and highest stage of AKI |
| Meersch, M.  2017 [3]  (PrevAKI) | 1, 276 | Adult patients after cardiac surgery requiring CPB with Nephrocheck > 0.3 | Efficacy KDIGO guidelines to prevent CSA-AKI in high risk patients defined by renal biomarkers | Preoperative sCr used as baseline, further according to KDIGO | Hourly urine output | Any AKI within 72 hours of cardiac surgery |
| Myles, P.S.  2018 [4]  (RELIEF) | 47, 2983 | Adults at risk of complications undergoing major abdominal surgery | Restrictive versus Liberal Fluid Therapy for disability free survival at 1 year | Did not estimate baseline sCr if this was missing, performed sensitivity analysis, sCr adjusted for FB, further according to KDIGO | Did not use urine output | Any AKI |
| AKI as selection criteria | | | | | | |
| Gaudry. S.  2016 [5]  (AKIKI) | 31, 619 | Adult ICU patients | Early vs. Late initiation of RRT and effect on survival on day 60 | Baseline sCr was value in the year previous admission or estimated using MDRD, , further according to KDIGO | Hourly urine output | KDIGO Stage 3 without potentially life-threatening complication directly related to renal failure |
| Zarbock, A.  2016 [6]  (ELAIN) | 1, 231 | Adult ICU patients | Early vs. Late initiation of RRT and effect on 90-day mortality | Baseline sCr was value in the last 3 months, at hospital admission or estimated using MDRD, further according to KDIGO | Hourly urine output | KDIGO Stage 2 |
| *Inclusion ongoing*  [7]  (STARRT-AKI) | 170, 2886  (planned) | Adult ICU patients | Timing of initiation of RRT and effect on 90-day mortality | Increase in sCr 2x > baseline, or sCr ≥354 μmol/L + evidence of a minimum increase of 27 μmol/L from baseline | Overall urine output less than 6ml/kg for the previous 12h | Severe AKI, absolute evidence of kidney dysfunction |
| *Inclusion ongoing*  [8]  (REVERSE AKI) | 7, 100  (planned) | Adult ICU patients | Fluid restrictive approach, compared to standard therapy for cumulative fluid balance at 72 hours | Increase in sCr 1.5x >baseline without a decline of 27umol/l or more from the last preceding measurement (at least 12 hours apart) | Overall urine output less than 0.5ml/kg/h for the previous 12h (with urine catheter in place for the period) | Moderate AKI (not requiring RRT at time of inclusion or expected RRT in the next 6 hours) |

*Description***:** Examples of randomized controlled trials that have used or are using AKI as either outcome or as selection criteria. *Abbreviations*: ICU = Intensive Care Unit, CPB = Cardio Pulmonary Bypass, AKI = Acute Kidney Injury, CSA = Cardiac Surgery Associated, sCr= serum Creatinine, RRT = Renal Replacement Therapy, KDIGO = Kidney Disease Improving Global Outcome.

**E-Table 2:** Different incidences of acute kidney injury according to 30 different methodological options in the same population of critically ill patients

| INCIDENCES OF AKI | | | | | | | | |  |
| --- | --- | --- | --- | --- | --- | --- | --- | --- | --- |
|  | **Variances in sCr (A)** | | | | | **Variances in UO (B)** | | | |
|  | **A1B2** (no sCr)  ***N = 989*** | ***A2B2***  ***N = 989*** | **A3B2  *N = 989*** | **A4B2**  ***N = 989*** | **A5B2**  ***N = 989*** | **A2B1** (no UO)  ***N = 1008*** | ***A2B2***  ***N = 989*** | **A2B3**  ***N = 989*** | **A2B4**  ***N = 728*** |
| C1, highest stage, KDIGO 0  % (95%CI) KDIGO 1  KDIGO 2  KDIGO 3 | 46 (43-49)  25 (23-28)  20 (18-23)  8 (7-10) | 38 (35-41)  29 (27-32)  22 (19-25)  11 (9-13) | 33 (30-36)  29 (26-32)  23 (21-26)  15 (13-18) | 32 (29-35)  27 (25-30)  24 (21-27)  17 (14-19) | 25 (23-28)  29 (27-32)  27 (25-30)  18 (16-20) | 72 (69-75)  14 (12-16)  5 (4-7)  9 (7-11) | 38 (35-41)  29 (27-32)  22 (19-25)  11 (9-13) | 46 (43-49)  29 (26-32)  15 (13-17)  10 (8-12) | 59 (57-63)  15 (12-17)  8 (6-10)  18 (15-20) |
| C2, any AKI, % (95%CI) | 54 (51-57) | 62 (59-67) | 67 (64-70) | 68 (65-71) | 75 (72-77) | 28 (25-31) | 62 (59-67) | 54 (51-58) | 43 (39-46) |
| C3, on both, % (95%CI) |  | 18 (16-21) | 24 (22-27) | 27 (24-30) | 33 (29-36) |  | 18 (16-21) | 16 (14-19) | 17 (14-20) |
| C4, severe, % (95%CI) | 29 (26-32) | 33 (30-36) | 39 (36-42) | 41 (38-44) | 45 (42-48) | 14 (12-16) | 33 (30-36) | 25 (22-28) | 26 (23-29) |

*Description:* All 30 variations of AKI and their incidence with 95% Confidence Intervals. Option A2B2 is shown in duplicate, for overview of variation in both sCr (A) and UO (B). A2B2: Only available baseline sCr, hourly six hour sliding windows of UO. A3B2: Using MDRD for missing baseline sCr, hourly six hour sliding windows of UO. A4B2: Using CKD-EPI for missing baseline sCr, hourly six hour sliding windows of UO. A5B2: Using age adjusted CKD-EPI for missing baseline sCr, hourly six hour sliding windows of UO. A2B1: Only available baseline sCr, without using UO. A2B3: Only available baseline sCr, hourly six hour fixed windows of UO. A2B4: Only available baseline sCr, 24 hour UO data averaged; see **figure 1**.

**E-Table 3:** Different incidences of acute kidney injury with more granular methods

|  | Incidences of AKI in more granular expressions | | | | | | | | |
| --- | --- | --- | --- | --- | --- | --- | --- | --- | --- |
|  | **A1B2**  (no sCr) | ***A2B2*** | **A3B2** | **A4B2** | **A5B2** | **A2B1**  (no UO) | ***A2B2*** | **A2B3** | **A2B4** |
| Persistent AKI, n (95%CI) | 22 (20-25) | 37 (34-40) | 30 (27-33) | 32 (29-35) | 35 (32-38) | 21 (19-24) | 37 (34-40) | 31 (28-34) | 26 (23-29) |
| Duration of AKI, median (IQR) | 0.20 (0.00-0.80) | 0.43 (0.00-1.00) | 0.50 (0.00-1.00) | 0.54 (0.00-1.00) | 0.86 (0.00-1.00) | 0.00 (0.00-0.33) | 0.43 (0.00-1.00) | 0.20 (0.00-1.00) | 0.00 (0.00-0.50) |
| AKI burden, median (IQR) | 0.05 (0.00-0.10) | 0.07 (0.00-0.22) | 0.05 (0.00-0.14) | 0.05 (0.00-0.15) | 0.07 (0.00-0.19) | 0.00 (0.00-0.14) | 0.07 (0.00-0.22) | 0.04 (0.00-0.17) | 0.00 (0.00-0.21) |

*Description:* More variations of AKI using more granular expressions of AKI. Option A2B2 is shown in duplicate, for overview of variation in both sCr (A) and UO (B). See description above, or see **figure 1** for abbreviated terms.

**E-Table 4**. Estimated and actual baseline creatinine

|  | N=449 |
| --- | --- |
| Creatinine baseline, median (IQR) | 76 (58, 102) |
| Estimation CKD EPI, GFR age adjusted, median (IQR) | 64 (48, 69) |
| Estimation MDRD, median (IQR) | 78 (71, 84) |
| Estimation CKD EPI, GFR not age adjusted, median (IQR) | 89 (65, 125) |

*Abbreviations*: MDRD: Modification of Diet in Renal Disease, CKD EPI: Chronic Kidney Disease Epidemiology Collaboration, GFR: Glomerular Filtration Rate.

**E-Table 5**. Missing data by calendar day

| Number of observations | Day 1 | Day 2 | Day 3 | Day 4 | Day 5 | Day 6 | Day 7 |
| --- | --- | --- | --- | --- | --- | --- | --- |
| Patients in ICU | 1010 | 880 | 616 | 416 | 317 | 254 | 217 |
| sCr, % missing data | 2 % | 2 % | 2 % | 3 % | 2 % | 2 % | 3 % |
| Hourly UO, % missing data | 7 % | 3 % | 3 % | 2 % | 3 % | 2 % | 1 % |
| 24 hour cumulative UO % missing data | 77 % | 50 % | 33 % | 32 % | 26 % | 27 % | 31 % |

*Abbreviations*: ICU: Intensive Care Unit, sCr: Serum creatinine, UO: Urine output.

**E-Table 6**. Univariable associations with 90-day mortality, odds ratios per AKI variation

|  | A1B2  (UO only) | A2B2 | A3B2 | A4B2 | A5B2 | A2B1  (sCr only) | A2B3 | A2B4 |
| --- | --- | --- | --- | --- | --- | --- | --- | --- |
| C1, highest stage | 1.39 (1.15-1.66, 0.58) | 1.43 (1.24-1.64, 0.59) | 1.44 (1.26-1.65, 0.60) | 1.47 (1.29-1.68, 0.61) | 1.37 (1.19-1.57, 0.59) | 1.58 (1.38-1.82, 0.59) | 1.52 (1.32-1.74, 0.60) | 1.38 (1.22-1.55, 0.59) |
| C2, any AKI | 1.35 (1.02-1.80, 0.54) | 1.45 (1.07-1.96, 0.54) | 1.74 (1.26-2.40, 0.56) | 1.79 (1.29-2.48, 0.56) | 1.48 (1.05-2.08, 0.54) | 2.11 (1.57-2.85, 0.58) | 1.61 (1.20-2.15, 0.56) | 2.16 (1.59-2.92, 0.60) |
| C3, on both | N/A | 2.02 (1.43-2.84, 0.56) | 2.19 (1.60-2.99, 0.58) | 2.14 (1.57-2.91, 0.58) | 1.77 (1.31-2.37, 0.56) | N/A | 2.20 (1.54-3.14, 0.56) | 2.85 (1.90-4.28, 0.59) |
| C4, severe AKI | 1.91 (1.42-2.58, 0.57) | 1.92 (1.44-2.57, 0.58) | 1.85 (1.39-2.46, 0.57) | 1.99 (1.50-2.65, 0.58) | 1.73 (1.30-2.29, 0.57) | 3.28 (2.27-4.73, 0.58) | 2.51 (1.85-3.41, 0.59) | 2.28 (1.68-3.09, 0.58) |

*Abbreviations*: AKI: Acute Kidney Injury, sCr: Serum creatinine, UO: Urine output. NA: Not applicable. Description: Displayed numbers are Odds Ratio’s (95%CI, AUROC below) for that type of AKI and 90-day mortality. . = non-significant association

|  | A1B2  (UO only) | *A2B2* | A3B2 | A4B2 | A5B2 | A2B1  (sCr only) | A2B3 | A2B4 |
| --- | --- | --- | --- | --- | --- | --- | --- | --- |
| Persistent AKI | 1.71 (1.41-2.01, 0.55) | 2.74 (2.05-3.67, 0.62) | 1.77 (1.32-2.39, 0.56) | 1.66 (1.23-2.22, 0.56) | 1.48 (1.11-1.97, 0.55) | 4.46 (3.14-6.32, 0.64) | 3.20 (2.38-4.31, 0.63) | 2.97 (2.19-4.03, 0.61) |
| Duration of AKI | . | . | 1.57 (1.13-2.18, 0.55) | 1.54 (1.11-2.14, 0.55) | . | 2.17 (1.53-3.09, 0.58) | 1.61 (1.16-2.24, 0.58) | 1.98 (1.41-2.78, 0.57) |
| AKI Burden | 6.41 (3.24-12.7, 0.57) | 8.72 (4.91-15.4, 0.60) | 7.54 (3.95-14.4, 0.61) | 7.65 (4.01-14.6, 0.61) | 5.96 (3.15-11.3, 0.58) | 6.86 (4.18-11.3, 0.63) | 10.1 (5.67-18.1, 0.62) | 8.19 (4.93-13.6, 0.63) |

**E-Table 7**. Univariable associations with 90-day mortality from more granular methods

*Abbreviations*: AKI: Acute Kidney Injury, sCr: Serum creatinine, UO: Urine output. Description: Displayed numbers are the Odds Ratio’s (95%CI, AUROC below for that type of AKI and 90-day mortality. . = non-significant association.


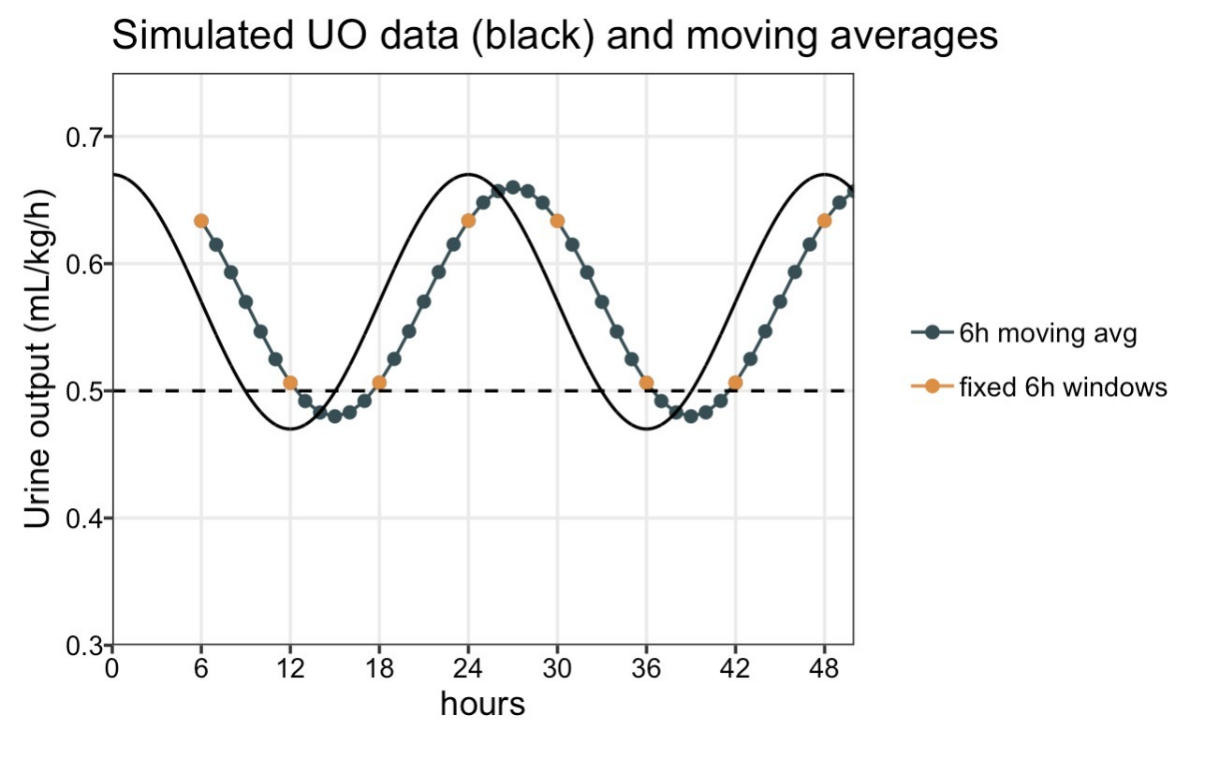
**E-Figure 1.** Example of using fixed versus sliding 6 hour windows vs actual (simulated) UO

*Description:* Black line represents simulated varying urine output per hour. Blue dots on the second line represents how using a sliding window may lead to AKI (when six dots are below the reference line of 0.5 mL/kg/h) and a fixed window could miss this episode (as in that window, perhaps only three dots are below the reference line).

**E-Figure 2.** Flowchart of study inclusion


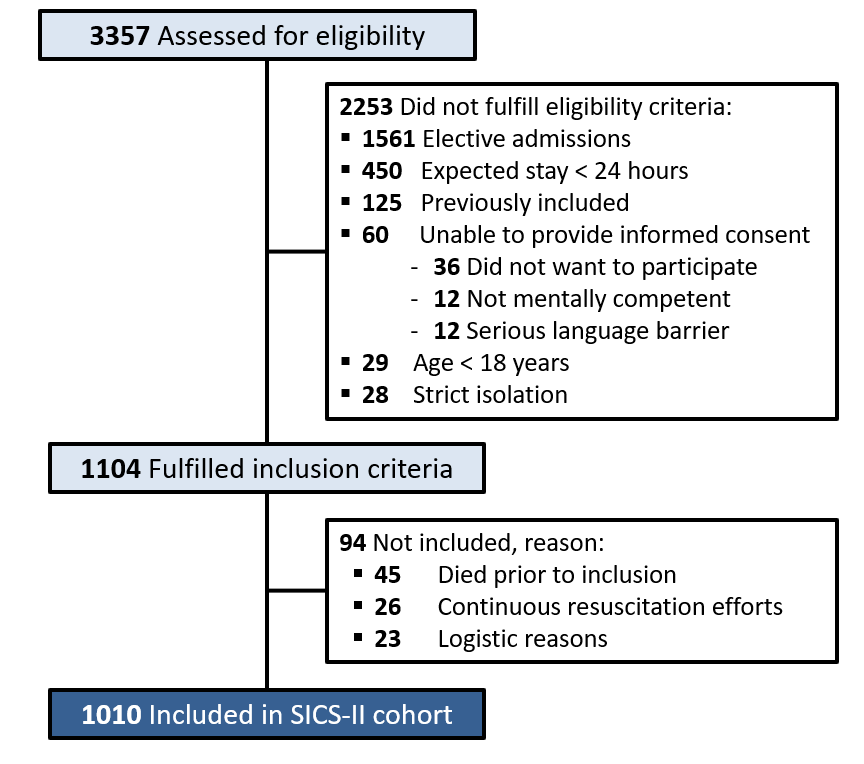


**E-Figure 3:** Variation in AKI incidence after excluding CKD patients


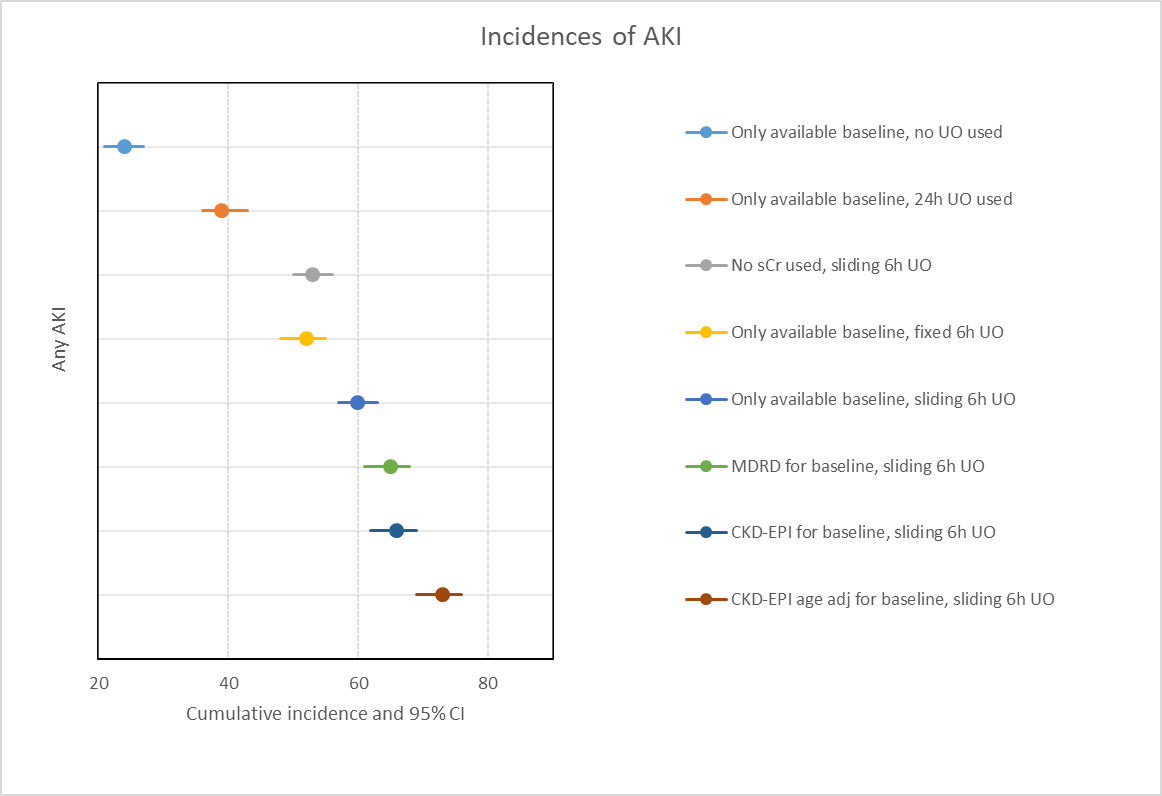


**E-Figure 4:** Variation in AKI incidence after excluding patients where no baseline sCr was known **
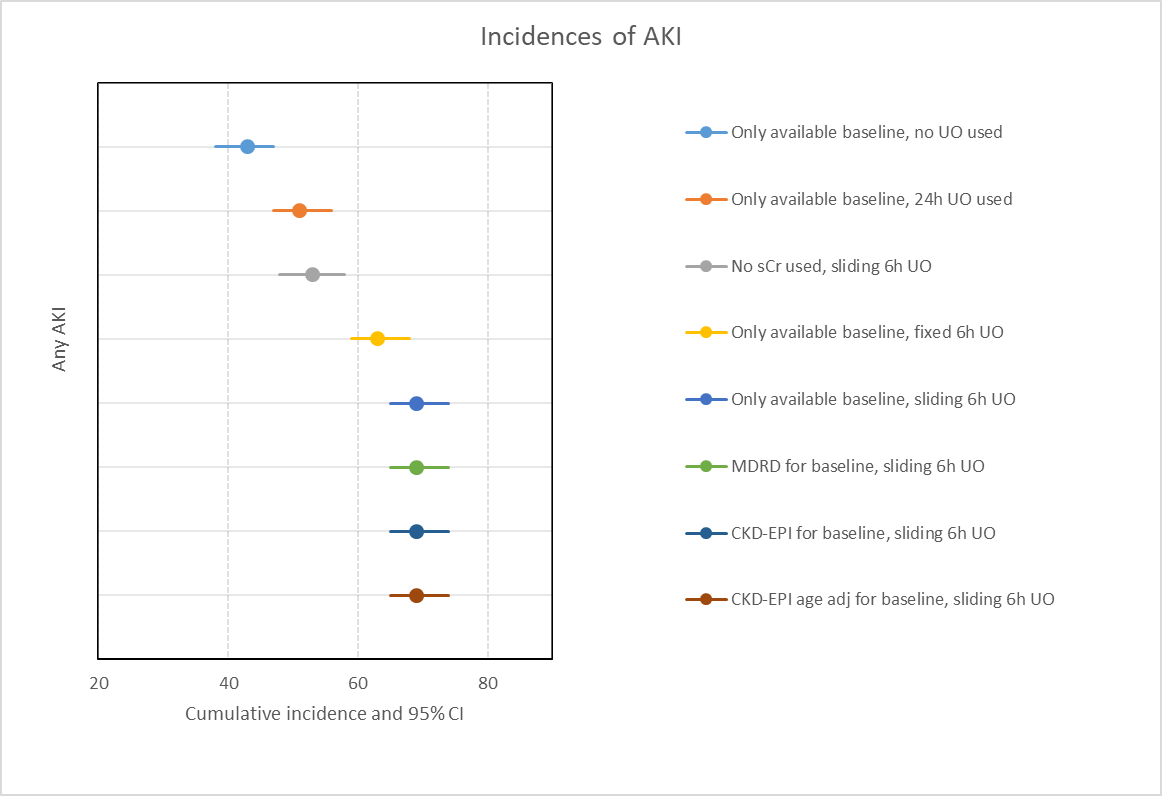
**

**References**

1. Young P, Bailey M, Beasley R, Henderson S, Mackle D, McArthur C, et al. Effect of a buffered crystalloid solution vs saline on acute kidney injury among patients in the intensive care unit: The SPLIT randomized clinical trial. JAMA - J Am Med Assoc. American Medical Association; 2015;314:1701–10.

2. Barba-Navarro R, Tapia-Silva M, Garza-Garcia C, López-Giacoman S, Melgoza-Toral I, Vázquez-Rangel A, et al. The Effect of Spironolactone on Acute Kidney Injury After Cardiac Surgery: A Randomized, Placebo-Controlled Trial. Am J Kidney Dis. W.B. Saunders; 2017;69:192–9.

3. Meersch M, Schmidt C, Hoffmeier A, Van Aken H, Wempe C, Gerss J, et al. Prevention of cardiac surgery-associated AKI by implementing the KDIGO guidelines in high risk patients identified by biomarkers: the PrevAKI randomized controlled trial. Intensive Care Med. Springer Verlag; 2017;43:1551–61.

4. Myles PS, Bellomo R, Corcoran T, Forbes A, Peyton P, Story D, et al. Restrictive versus liberal fluid therapy for major abdominal surgery. N Engl J Med. Massachussetts Medical Society; 2018;378:2263–74.

5. Gaudry S, Hajage D, Schortgen F, Martin-Lefevre L, Pons B, Boulet E, et al. Initiation Strategies for Renal-Replacement Therapy in the Intensive Care Unit. N Engl J Med. United States; 2016;375:122–33.

6. Zarbock A, Kellum JA, Schmidt C, Van Aken H, Wempe C, Pavenstädt H, et al. Effect of early vs delayed initiation of renal replacement therapy on mortality in critically ill patients with acute kidney injury: The elain randomized clinical trial. JAMA - J Am Med Assoc. American Medical Association; 2016;315:2190–9.

7. STandard versus Accelerated initiation of Renal Replacement Therapy in Acute Kidney Injury: Study Protocol for a Multi-National, Multi-Center, Randomized Controlled Trial. Can J Kidney Heal Dis. SAGE Publications Ltd; 2019;6.

8. REVERSE-AKI Randomized Controlled Pilot Trial - Full Text View - ClinicalTrials.gov [Internet]. [cited 2019 Dec 5]. Available from: https://clinicaltrials.gov/ct2/show/NCT03251131
